# *Candidatus* Cryptoplasma Associated with Green Lizards and *Ixodes ricinus* Ticks, Slovakia, 2004–2011

**DOI:** 10.3201/eid2412.161958

**Published:** 2018-12

**Authors:** Božena Kočíková, Igor Majláth, Bronislava Víchová, Lenka Maliničová, Peter Pristaš, Vincent A. Connors, Viktória Majláthová

**Affiliations:** Slovak Academy of Sciences, Košice, Slovakia (B. Kočíková, B. Víchová, P. Pristaš, V. Majláthová);; Pavol Jozef Šafárik University in Košice, Košice (I. Majláth, L. Maliničová, P. Pristaš, V. Majláthová);; University of South Carolina Upstate, Spartanburg, South Carolina, USA (V.A. Connors)

**Keywords:** *Anaplasmataceae*, reptile-associated *Candidatus* Cryptoplasma, *Lacerta viridis*, green lizard, *Ixodes ricinus*, Slovakia, Karst, tick-borne infections, zoonoses, green lizards, ticks, Slovak Karst National Park, bacteria

## Abstract

During 2004–2011, we collected green lizards and *Ixodes ricinus* ticks in Slovak Karst National Park in Slovakia; 90% (36/40) of lizards and 37% of ticks removed from lizards were infected with family *Anaplasmataceae* bacteria. Only *Candidatus* Cryptoplasma sp. REP (reptile) was identified in these samples. Green lizards transmit this bacterium.

The family *Anaplasmataceae* (Rickettsiales; Alphaproteobacteria) comprises bacteria that are able to invade and infect their vertebrate host’s blood cells, bone marrow–derived phagocytic cells, and endothelial cells; these bacteria can also infect cells of insects, helminths, and arthropod reproductive tissues (*1*–*3*). Tickborne family members include bacteria of *Anaplasma*, *Ehrlichia*, *Candidatus* Neoehrlichia sp., and *Candidatus* Cryptoplasma californiense (*4*).

Although reptiles play a role as hosts for ixodid and argasid ticks, their role in maintaining tickborne *Anaplasmataceae* bacteria in the environment has not been described. Nieto et al. (*5*) suggested that lizards and snakes in the far western part of the United States could become exposed to *Anaplasma phagocytophilum* when fed on by infected ticks. Moreover, Rejmanek et al. detected 2 highly dissimilar strains of *A. phagocytophilum* in the same lizard species (*6*). In Europe, an undescribed *Anaplasma* sp. was detected in *Ixodes ricinus* ticks feeding on sand lizards and sand lizard blood samples (*7*,*8*). In our study, we sought to confirm these previous findings by determining whether family *Anaplasmataceae* bacteria were present in lizards and their feeding ticks in Slovakia.

## The Study

We conducted this study in the Slovak Karst National Park in Slovakia (48°36′N, 20°52′E) during 2004–2011. We carried out lizard capture and sample collections with official permits (6103/2007-2.1 and 5498/2011-2.2) issued by the Ministry of Environment of the Slovak Republic. We captured 103 green lizards (*Lacerta viridis*) and collected blood from 40 (30 males and 10 females). We collected 235 *I. ricinus* ticks (118 larvae and 117 nymphs) from 63 green lizards and 271 questing *I. ricinus* ticks (132 nymphs, 76 males, and 63 females) from the same area and immediately stored them in 70% ethanol.

We isolated DNA from lizard blood using a DNeasy Blood & Tissue *Kit* (QIAGEN, Hilden Germany) and isolated DNA from ticks by alkaline hydrolysis. We performed PCR amplification in 25-μL (total) reaction mixtures using the Master*Taq* DNA Polymerase Kit (Eppendorf AG, Hamburg, Germany). We amplified sequences using the primer combinations EHR747 plus EHR521 or fD1 plus rP2 (*9*), which spanned almost the entire 16S rRNA sequence (Table 1). We examined the ≈250-bp gene fragment of 16S rRNA by single-strand conformation polymorphism (SSCP) analysis to determine *Anaplasmataceae* species type (*10*). We performed SSCP analysis following the protocol of Derdakova et al. (*11*). We ran positive control samples *A. phagocytophilum*, *A. ovis*, *Wolbachia* sp., and *Candidatus* N. mikurensis with each reaction. We purified the PCR products obtained using the GenElute PCR Clean-Up Kit (Sigma-Aldrich, Buchs, Switzerland) and sequenced both strands. We edited variants obtained in this study (1,410 bp) using MEGA 4.0.2 (https://megasoftware.net/) and checked by eye. We made comparisons to sequences in GenBank with BLASTn 2.2.26 (https://pods.iplantcollaborative.org/wiki/display/DEapps/Blastn-2.2.26). For phylogenetic analysis of our variant (GenBank accession no. MG924904), we aligned 17 related sequences obtained from the GenBank database and constructed a phylogenetic tree using the Bayesian inference method (*12*).

**Table 1:** Primers used to amplify 16S rRNA gene of *Candidatus* Cryptoplasma sp. found in green lizards and *Ixodes ricinus* ticks, Slovakia, 2004–2011

Organism	Primer name	Sequences, 5′ → 3′	Length of amplified fragment, bp	Reference
Family *Anaplasmataceae*	EHR747	GCACTCATCGTTTACAGCGTG	247	(*10*)
	EHR521	TGTAGGCGGTTCGGTAAGTTAAAG		
Most eubacteria	fD1	ccgaattcgtcgacaacAGAGTTTGATCCTGGCTCAG	1,500	(*9*)
	rP2	cccgggatccaagcttACGGCTACCTTGTTACGACTT		

We examined all blood and ticks collected for the presence of family *Anaplasmataceae* bacteria. *Anaplasmataceae* family members were present in 36 lizards (28 males and 8 females). Of the ticks removed from lizards, 87 (37%) were infected, and of questing ticks, 18 (6.6%) were infected (Table 2).

**Table 2:** Prevalence of family *Anaplasmataceae* bacteria in *Ixodes ricinus* ticks collected from green lizards and surrounding vegetation, Slovakia, 2004–2011

Tick source, type	No. ticks examined	No. (%) positive ticks	No. (%) positive ticks			
			*Candidatus* Cryptoplasma	*Anaplasma phagocytophilum*	*Wolbachia pipientis*	*Candidatus* Neoehrlichia mikurensis
Lizards						
Larvae	118	43 (36.4)	43 (100)	–	–	–
Nymphs	117	44 (37.6)	44 (100)	–	–	–
Total	235	87 (37)	87 (100)	–	–	–
Vegetation						
Nymphs	132	8 (6.1)	2	3	3	0
Males	76	4 (5.3)	1	2	0	1
Females	63	6 (9.5)	3	2	1	0
Adults	139	10 (7.2)	4	4	1	1
Total	271	18 (6.6)	6	7	4	1

**Figure 1:**
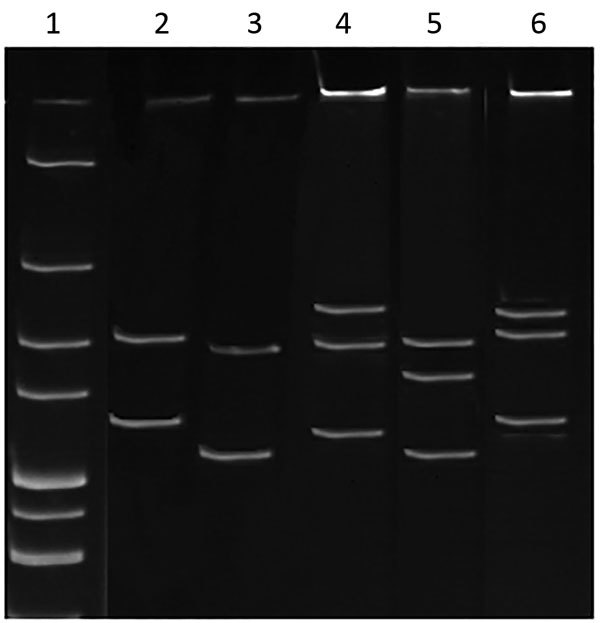
Single-strand conformation polymorphism profile of *Anaplasmataceae* isolate from reptiles, Slovakia, 2004–2011. The 247-bp 16S rRNA PCR fragments from the isolate from reptiles and known *Anaplasmataceae* species were denatured and electrophoresed. Lane 1, 100-bp ladder marker; lane 2, *Candidatus* Neoehrlichia mikurensis; lane 3, *Anaplasma phagocytophilum*; lane 4, isolate *Candidatus* Cryptoplasma sp. REP (reptile) obtained in this study; lane 5, *A. ovis*; and lane 6, *Wolbachia*.

**Figure 2:**
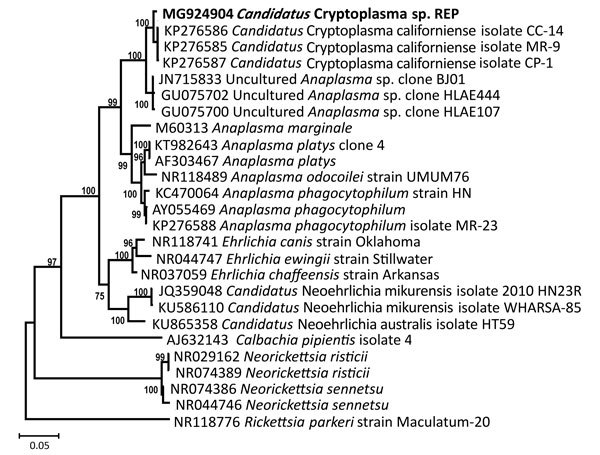
Phylogenetic relatedness of *Candidatus* Cryptoplasma sp. REP (reptile; bold), Slovakia, 2004–2011, to other *Anaplasmataceae* sp. family members. We constructed the tree using 16S rRNA sequences and the Bayesian inference method. The *Rickettsia parkeri* sequence was used as an outgroup. Scale bar indicates nucleotide substitutions per site.

Denatured and electrophoresed PCR products from samples demonstrated several SSCP profiles, of which 1 was clearly distinguishable from the profiles of the *Anaplasmataceae* species used as controls (Figure 1). We detected this unique profile in all lizard blood samples, all ticks feeding on lizards, and some questing ticks. We sequenced representatives of this unidentified SSCP profile (≈247 bp; GenBank accession nos. KY031322–3) and compared them with DNA fragments in the GenBank database. The closest related (99% identity) 16S rRNA sequences were from uncultured *Anaplasma* sp. isolates from questing *I. ricinus* ticks from Morocco (GenBank accession no. AY672415), Tunisia (GenBank accession no. AY672420), and France (GenBank accession no. GU734325). Sequencing of a longer (1,410-bp) fragment of the 16S rRNA gene revealed 99.1% similarity with the *Candidatus* C. californiense isolate from *I. pacificus* ticks in California (Figure 2). The 16S rRNA sequence obtained in this study was found to share a maximum of 94% identity with *A. phagocytophilum* Norway variant 2 (GenBank accession no. CP015376). The phylogenetic tree we constructed using 16S rRNA gene sequences showed that the reptile-associated *Candidatus* Cryptoplasma sp. REP (reptile) clustered in a separate branch with *Candidatus* C. californiense, indicating the isolate represents a lineage distinct from other known *Anaplasmataceae* species (e.g., *A. phagocytophilum*, *A. marginale*, *A. platys*, *Ehrlichia muris*, *E. chaffeensis*, and *E. ewingii*).

## Conclusions

The role of ectotherm animals, especially lizards, in the maintenance of vectorborne pathogens is not clear. The interaction between reptiles and *Anaplasmataceae* family members has only been investigated in a few studies. Our findings expand knowledge on this research topic. Only limited information about the reptile–*Anaplasma* relationship exists. Ekner et al. suggested that sand lizards could potentially serve as a reservoir host for species of the *Anaplasmataceae* family when she discovered that ticks collected from these lizards in Poland were infected with *Anaplasma*-like pathogens (*8*). Although *A. phagocytophilum* might be transmitted by reptiles to a limited extent (*5*), the *Anaplasma*-like species detected in reptiles could also be a novel species, as suggested by Rejmanek et al. (*6*).

Despite the fact that lizards are exposed to a number of family *Anaplasmataceae* bacteria through infected ticks, our findings suggest that, except for *Canditatus* Cryptoplasma sp. REP, green lizards do not acquire infections with these species. In short, we detected *Canditatus* Cryptoplasma sp. REP in 90% of examined lizards, 37% of ticks feeding on lizards, and 6.6% of questing ticks in localities with lizards.

On the basis of our results, we cautiously speculate that *Canditatus* Cryptoplasma sp. REP is selected for and other genospecies selected against in ticks feeding on lizards. The *Canditatus* Cryptoplasma sp. REP variant had a high homology (100%) with a sequence obtained from an *Apodemus agrarius* mouse from Slovakia (*13*), which indicates that rodents or other mammals might also become infected with this bacterium and contribute (to a lesser extent) to the circulation of these bacteria in nature.

In conclusion, we found a yet to be named species of *Canditatus* Cryptoplasma sp. (*Canditatus* Cryptoplasma sp. REP) in questing *I. ricinus* ticks, *I. ricinus* ticks collected from and feeding on green lizards, and the blood of green lizards in Slovakia. These results indicate that green lizards serve as an intermediate host for this bacterium and that lizards can influence the enzootic maintenance and circulation of bacteria in the environment. However, other hosts besides reptiles could be involved in the *Canditatus* Cryptoplasma sp. REP lifecycle as well, though probably to a lesser extent.
